# Developing a Novel Terahertz Fabry–Perot Microcavity Biosensor by Incorporating Porous Film for Yeast Sensing

**DOI:** 10.3390/s23135797

**Published:** 2023-06-21

**Authors:** Hwan Sik Kim, Seung Won Jun, Yeong Hwan Ahn

**Affiliations:** Department of Physics and Department of Energy Systems Research, Ajou University, Suwon 16499, Republic of Korea; ktj1214@naver.com (H.S.K.); sunsoon12382@gmail.com (S.W.J.)

**Keywords:** Fabry–Perot cavity, microorganisms, porous film

## Abstract

We present a novel terahertz (THz) Fabry–Perot (FP) microcavity biosensor that uses a porous polytetrafluoroethylene (PTFE) supporting film to improve microorganism detection. The THz FP microcavity confines and enhances fields in the middle of the cavity, where the target microbial film is placed with the aid of a PTFE film having a dielectric constant close to unity in the THz range. The resonant frequency shift increased linearly with increasing amount of yeasts, without showing saturation behavior under our experimental conditions. These results agree well with finite-difference time-domain (FDTD) simulations. The sensor’s sensitivity was 11.7 GHz/μm, close to the optimal condition of 12.5 GHz/μm, when yeast was placed at the cavity’s center, but no frequency shift was observed when the yeast was coated on the mirror side. We derived an explicit relation for the frequency shift as a function of the index, amount, and location of the substances that is consistent with the electric field distribution across the cavity. We also produced THz transmission images of yeast-coated PTFE, mapping the frequency shift of the FP resonance and revealing the spatial distribution of yeast.

## 1. Introduction

Terahertz (THz) spectroscopy has shown great potential as a label-free, non-contact, and non-destructive technique for sensing chemical and biological substances [1,2,3,4,5,6,7,8,9,10,11,12,13,14,15,16,17,18,19]. Metamaterials operating in the THz range are particularly useful for detecting biological substances, including microorganisms and viruses [20,21,22,23,24,25,26,27,28,29,30,31,32]. Recently, novel label-free sensing tools using thermal curve analysis have been introduced, where the metamaterial sensor resonance is monitored as a function of temperature [33]. The dielectric constant of the microbial film changes at the transition temperature between multiple stages of their growth and death phases, enabling label-free sensing. To enhance the sensitivity of microbial sensing, various metamaterial designs have been proposed, such as by varying geometrical parameters like the gap width, substrate index, and gap functionalization [34,35,36]. In particular, in metamaterial sensing, the effective sensing area is strongly localized in the gap area, therefore, microbes located outside the gap area do not contribute to sensing [37]. Consequently, localizing the target substances toward the gap area is essential to improve sensitivity; various attempts have been made, including the implementation surface functionalization, electrophoresis, and physical sweeping techniques [37,38,39].

Conversely, the effective volume can be markedly improved by introducing microcavity structures, such as Fabry–Perot (FP) interferometers. FP interferometers serve as key components in the development of large-area biosensors owing to their geometric simplicity and their ability to confine light between parallel reflective surfaces [40,41,42,43,44,45,46]. The fabrication of these optical resonators does not require lithography, allowing for cost-effective and scalable biosensor implementation [47,48]. In contrast to metamaterial sensing, the effective volume can be improved substantially by the insertion of a microbial layer in the middle of the FP interferometer because the entire film contributes to sensing by introducing microcavity structures [49,50]. Importantly, the lowest resonant mode is preferred because it exhibits higher sensitivity when the field is localized in a single domain [49,50]. The presence of substances other than the target material inside the FP cavity lowers the sensitivity of the sensor. Therefore, the introduction of a supporting film with a dielectric constant close to unity is critical to avoid disturbing the field distribution (i.e., enhancement effects) inside the cavity. In the THz frequency range, FP microcavity has been used for sensitive sensing of gases and liquids; conversely, it has not been considered as a biosensing platform [51,52,53,54,55,56,57,58,59,60]. A careful consideration of the spatial field distribution inside the microcavities is crucial to achieve biological sensing using FP microcavities.

In this study, we developed a biosensing platform using a THz FP microcavity. For enhanced sensitivity, the microcavity was incorporated with a porous supporting film with a dielectric constant close to unity in the THz range. We performed THz time-domain spectroscopy to verify the usefulness of the FP cavity sensor on yeast cells and characterized its sensitivity. We performed finite-difference time-domain (FDTD) simulations and successfully reproduced these observations. Finally, we performed transmission imaging of the FP cavity with the yeast film and obtained a distribution image of the yeast film.

## 2. Device Fabrication and Experimental Setup

**Figure 1:**
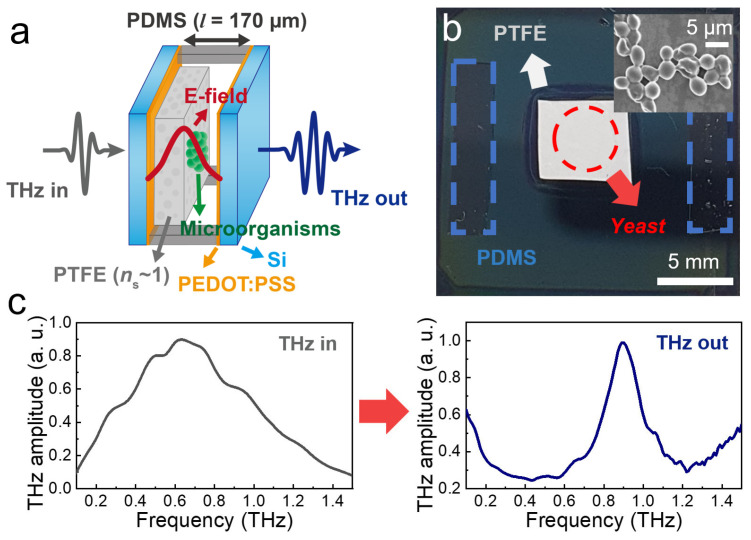
(**a**) Schematic illustration of the FP cavity with the yeast film located in the middle of the cavity. (**b**) Photograph of the partial mirror of the FP cavity. The red dashed line indicates the presence of yeast film on the PTFE membrane. (inset) Scanning electron microscopy image of the yeast cells. (**c**) Transmission spectra of input THz wave (**left**) and output wave from FP device with *l* = 170 μm without a yeast film (**right**).

In Figure 1, we depict our FP cavity sensor with a target material inserted at the center of the cavity using a porous supporting film. This is because a strong THz field is present at the center of the cavity for its fundamental mode. In this work, we introduced a polytetrafluoroethylene (PTFE) membrane with a high porosity of 80%, a pore size of 1.0 μm, and a thickness of 85 μm (JAWP04700, Merck KGaA, Darmstadt, Germany). PTFE membrane exhibits a dielectric constant close to unity (*n*_s_ ~ 1.07) at the THz frequency range due to its porous structure. By introducing a PTFE supporting film, the sensitivity of the FP cavity sensor can be optimized because the enhancement in the THz field in the middle of the cavity reaches its maximum value when the supporting film index is unity. This will be shown later in detail. In contrast, in most previous studies, the target materials filled the entire cavity volume, which could result in decreased sensitivity (i.e., frequency shift) and a reduction in transmission [43,44,45].

We fabricated a partial mirror of the FP cavity by spin-coating PEDOT: PSS (PH1000, 1000 rpm) twice on the high resistivity silicon substrate (>1000 Ω), which delivered transmission amplitudes of 35%. In this study, we doped PEDOT: PSS with ethylene glycol to improve film conductivity. The FP microcavity was fabricated by folding two partial mirrors. The cavity length (i.e., the distance between the two mirrors) was maintained at *l* = 170 μm, which corresponded to a resonant frequency of 0.87 THz. The cavity thickness was twice that of the PTFE membrane with a thickness of 85 μm, so that the target substances coated on the membrane were placed in the middle of the cavity. A polydimethylsiloxane (PDMS) mold was used as the spacer to maintain the cavity length, as shown in Figure 1a. We confirmed that the resonant frequency did not change during multiple folding and unfolding processes of the partial mirrors, which were required to locate the target substances in the middle of the cavity.

To prove the usefulness of the FP sensors, we tested them with yeast (*Saccharomyces cerevisiae*; KCTC 27139) grown by streaking on the culture medium of the Korean Agricultural Culture Collection (KACC) for two days. The culture media and the incubation temperature used for yeast growth were glucose-peptone-yeast extract agar and 25 °C, respectively [8]. They were dispersed in an aqueous solution and transferred to a PTFE membrane using the drop-casting method, as shown in Figure 1b. In other words, we dropped 10 μL droplets of a 7.9 mg/mL yeast solution onto the PTFE membrane, and repeated it five times. The droplets were then dried on a hot plate at 35 °C for 15 min, resulting in the yeast film thickness (*d*_yeast_) of 2.3 μm. The thickness was measured by using an alpha-step equipment. We repeated the same procedures to prepare thicker yeast films up to *d*_yeast_ = 10.1 μm. The scanning electron microscopy image of the yeast cells is shown as an inset. The yeast-coated membrane was attached to one of the partial mirrors by slightly wetting the mirror–membrane interface; it was covered by another partial mirror for the FP cavity.

THz transmission amplitudes of the THz cavity devices were obtained from a home-built THz time-domain spectroscopy (TDS) system based on a Ti:Sapphire laser [61]. The photoconductive antenna emits a linearly polarized THz pulse when a femtosecond laser (λ = 800 nm) is incident on it. We focused a THz pulse with a spot diameter of approximately 1 mm on the FP cavity at ambient conditions. Finally, THz spectra were obtained using Fourier transformation of the phase-sensitive amplitudes in the THz-TDS measurement, with which we recorded the THz spectra through the FP devices with a yeast film in the middle of the cavity. Representative spectra of the input THz pulse and the output pulse from the FP cavity device (in the absence of yeast layer) are illustrated in Figure 1c.

## 3. Results

**Figure 2:**
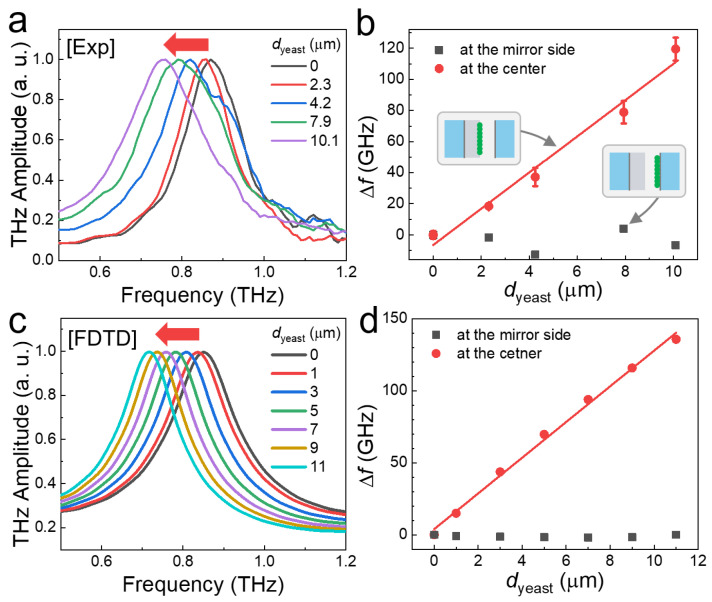
Resonance shift for varying film thicknesses. (**a**) Transmission amplitudes through the FP cavity device, for varying yeast layer thicknesses (*d*_yeast_) when the yeast layer is placed at the center of the cavity. (**b**) Plot of the frequency shift as a function of *d*_yeast_ when the yeast layer is placed at the center (red circles) and at the mirror side (black squares). The error bar indicates the standard deviation obtained from five different samples for each *d*_yeast_. The red solid line represents a linear fit to the data. (Insets) Schematic for the vertical location of yeast layers (depicted by green circles) inside the cavity. (**c**) FDTD simulation results for transmission amplitudes. (**d**) FDTD results for the frequency shift as a function of *d*_yeast_ when the yeast layer is placed at the center (red circles) and at the mirror side (black squares).

The FP resonance was recorded as a function of *d*_yeast_, as illustrated in Figure 2. The yeast solution was dropped onto the PTFE membrane, and the THz transmission spectra through the FP cavity were obtained with different *d*_yeast_ values, as shown in Figure 2a. The black line represents the resonant frequency of the cavity without yeast coating, while a strong resonance was found at 0.87 THz with a cavity length of *l* = 170 μm. A significant red shift was observed with increasing yeast layer thickness, in accordance with the relationship *f*_m_ = (*c*/*2l*_opt_)*m*, where *c* is the speed of light, *l*_opt_ is the optical path length in the cavity, and *m* is the cavity mode (in this case, *m* = 1 for the fundamental mode). When the cavity is filled with air, *l*_opt_ represents the physical distance between the mirrors. Conversely, in the presence of the yeast layer, the optical path length inside the cavity increases, resulting in a shift in the resonance. In other words, the resonance frequency exhibits a significant redshift because of the change in the dielectric configuration in the middle of the FP cavity.

The frequency shift (Δ*f*) was plotted as a function of *d*_yeast_ in Figure 2b (red circles); it increased linearly with the yeast film thickness, from 18 GHz to 119 GHz, as we increased *d*_yeast_ from 2.3 μm to 10.1 μm. We averaged the peak frequency values from five different samples for each *d*_yeast_. The sensitivity (*S*) was defined as the slope of the data in Figure 2b, leading to the equation *S* = Δ*f*/*d*_yeast_. By fitting the data (represented by the solid red line in Figure 2b), we obtained *S* = 11.7 GHz/μm. For dielectric sensing, GHz/RIU is a common unit to represent the sensitivity. In this case, the sensitivity was 11.7 GHz/μm/RIU using the refractive index of yeast film (*n*_yeast_ = 2.0) [8]. Conversely, when the yeast film was coated on the partial mirror (i.e., not in the middle of the cavity), no noticeable change in the resonant frequency was observed, as shown in Figure 2b (black squares). This confirms that the target substances should be located at the center of the cavity, where the THz field is localized and enhanced. The use of a porous supporting film plays a crucial role in sensing target substances, such as microorganisms with microcavity structures.

The sensitivity of the FP cavity is comparable to that of metamaterials. However, Δ*f* did not exhibit any saturation behavior even with a thickness of more than 10 μm. Therefore, a direct comparison of metamaterial sensitivity is limited. The sensitivity of a THz-metamaterial on a quartz substrate in terms of RIU was found to be 250 GHz/RIU, which is three times higher than that on a Si substrate (81 GHz/RIU) [62]. In this work, we extracted the sensitivity of 118 GHz/RIU at a thickness of 10 μm of the target material. Importantly, we note that the sensitivity of FP devices will increase further as the amount of specimen increases, whereas that of metamaterial sensor is saturated at 10 μm. This is in contrast with that of metamaterial sensing, in which Δ*f* saturates at a substance thickness of 2−3 μm, depending on the gap size [20].

To confirm our experimental observations, we performed an FDTD simulation (CST MICROWAVE STUDIO) using the parameters for the cavity geometry and yeast film [8,63]. The simulation successfully reproduced our experimental observations. Figure 2c shows the transmission amplitudes for different yeast film thicknesses when they were located in the middle of the cavity. The frequency shift as a function of the film thickness is plotted in Figure 2d (red circles). By fitting the data with a linear curve, we extracted a sensitivity of 12.4 GHz/μm (with a standard error of 0.4 GHz/μm), consistent with the experimental value of 11.7 GHz/μm (with a standard error of 1.0 GHz/μm). Conversely, no frequency shift was observed when the film was placed on one side of the partial mirror (black squares), as in the experiments (Figure 2b). This confirms the importance of field localization and enhancement inside the cavity for optimal sensitivity.

**Figure 3:**
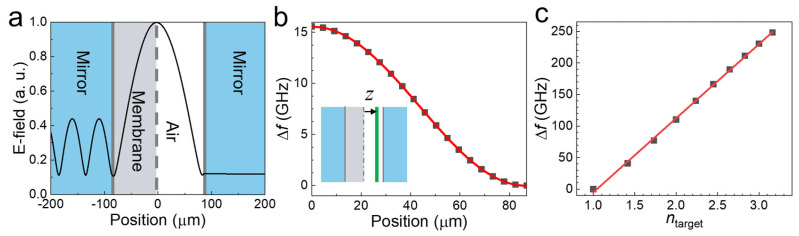
FDTD simulation results for different substance locations. (**a**) Simulated THz electric field (*E*_x_) as a function of the position in the cavity at 0.87 THz. (**b**) Resonant frequency shift as a function of dielectric film location (*z*). The solid line is a fit to the data. (**c**) Resonant frequency shift as a function of *n*_target_ for *z* = 0.

In addition, we explored the explicit relationship between the sensitivity (i.e., the frequency shift) and sensing parameters, such as the amount and the index of the target substances. Importantly, we found the explicit relation between Δ*f* and the location (*z*) of the microbial substances, which strongly influences Δ*f* in FP sensing. Figure 2b clearly demonstrates that Δ*f* is significantly suppressed when the yeast sample is located on the mirror side. This is because the strength of the electric field has a strong influence on Δ*f* as mentioned before. Figure 3a shows the distribution of the THz electric field in the cavity obtained from the FDTD simulation results, clearly indicating that the electric field was stronger in the central part of the cavity (i.e., at *z* = 0) than at the mirror side. We also plotted Δ*f* as a position function of *z* of the yeast layer (with a thickness of 1 μm) in Figure 3b and discovered that it exhibited a sinusoidal relationship of $\Delta f\left(z\right)\propto \cos^{2}\left(kz\right)$, with a constant *k* obtained at 0.018 rad/μm. This point has not been explicitly investigated before; in general, the target materials filled the entire cavity volume in most previous microcavity experiments. Additionally, we found that Δ*f* is proportional to the thickness of target substances (*d*_target_), as shown both experimentally and theoretically in Figure 2d. We also found that Δ*f* depends linearly on the refractive index of the target materials (*n*_target_) within our experimental conditions, as shown in Figure 3c. Consequently, we propose the resonance Δ*f* of the FP cavity sensor as a function of the thickness, refractive index, and position: $\Delta f\left(n_{target},d_{target},z\right)=A(n_{target}-1)d_{target}\cos^{2}\left(kz\right)$, where, *A* = 12.9 GHz/μm.

**Figure 4:**
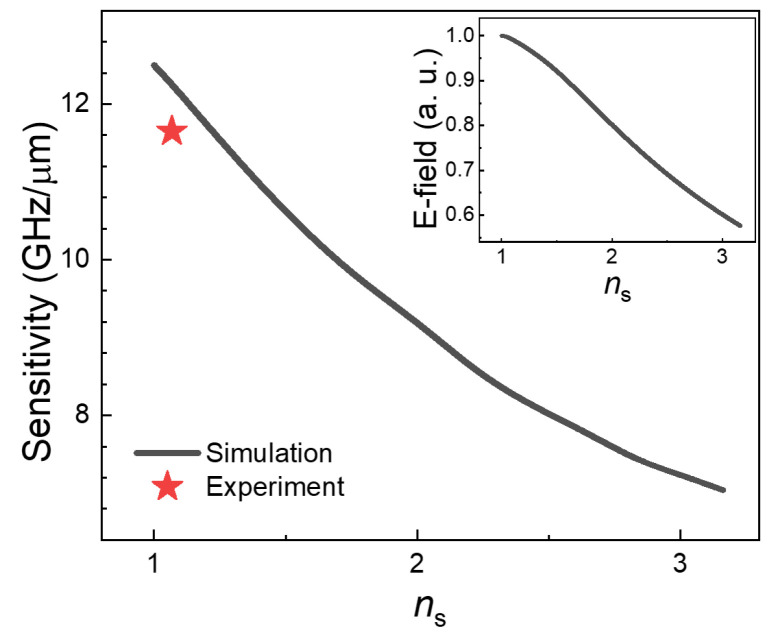
Sensitivity as a function of supporting film indices (*n*_s_) from 1 to 3.2. (inset) Peak electric field (*E*_x_) at 0.9 THz in the cavity without the yeast film.

As mentioned previously, the use of supporting films (such as PTFE films) is inevitable to locate the target substance toward the middle of the cavity. We performed FDTD simulations to address the effect of the supporting film index (*n*_s_) on sensitivity. The results are shown in Figure 4, in which we plotted the sensitivity, in terms of *S* = Δ*f*/*d*_yeast_, as a function of *n*_s_. As expected, the sensitivity decreased as *n*_s_ increased from 12.5 GHz/μm (for *n*_s_ = 1) to 7.0 GHz/μm (for *n*_s_ = 3.2). The sensitivity of the FP cavity sensor was the maximum when *n*_s_ was unity, which was slightly higher than that of our experiments using the PTFE membrane (11.7 GHz/μm, depicted by a red star). In other words, our device sensitivity was very close to the optimized conditions, in which there was no supporting film. This tendency can be understood by the decrease in field strength with increasing supporting film index, as shown in the inset of Figure 4.

**Figure 5:**
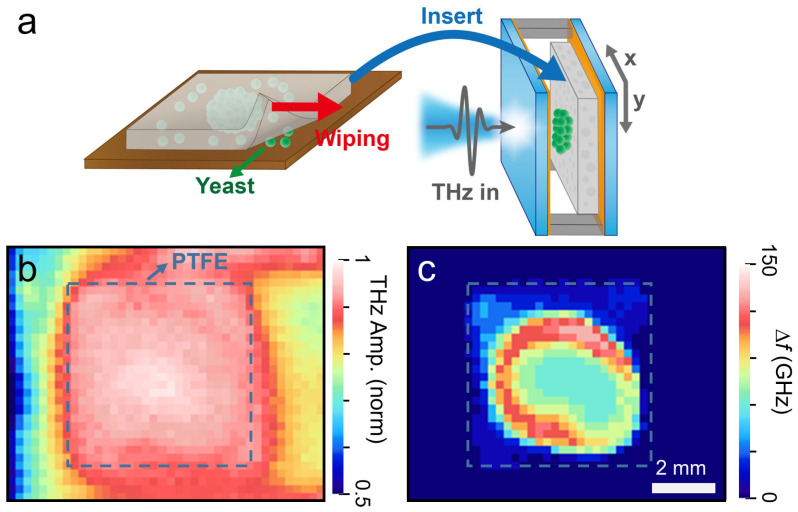
C-scan images for yeast film thickness. (**a**) Schematic illustration for the preparation of THz transmission imaging, (**b**) C-scan images for THz transmission amplitude through the sample with the yeast coated on PTFE. The dashed area indicates the PTFE region. (**c**) C-scan image for Δ*f* exhibiting the spatial distribution of the yeast layer. Δ*f* of 150 GHz corresponds to *d*_yeast_ of 12.8 μm.

Finally, we recorded THz transmission imaging on our FP device to obtain the spatial distribution of the microbes, as shown in Figure 5. We transferred the microbial layers dispersed on a glass substrate by wiping them with a PTFE film, which was subsequently placed in the FP cavity for THz measurements (Figure 5a). Figure 5b shows the THz transmission amplitude as a function of the lateral position (C-scan) with a scan range of 10 × 8 mm^2^ and a pixel size of 40 × 32 pxl^2^. The focal length was 5 cm and the spot size was 1 mm. The dashed line indicates the location of the PTFE membrane. However, it was difficult to distinguish the location of the yeast layer. Conversely, we could clearly identify the yeast layers in Figure 5c, where we plotted Δ*f* as a function of the THz spot position. Δ*f* exhibits a significant redshift owing to the change in the dielectric configuration in the middle of the FP cavity, as mentioned previously. Therefore, the image contrast in Figure 5c represents the amount of yeast coated on the membrane. Using the explicit relation for Δ*f* versus *d*_yeast_ as shown above, we could obtain the spatial distribution of yeast layers in a quantitative manner. For instance, Δ*f* of 150 GHz corresponds to *d*_yeast_ of 12.8 μm in Figure 5c. Therefore, THz imaging with FP microcavities can be a very useful tool for obtaining the spatial distribution of microbes and pathogens, which will help identify hazardous pathogens in real life. The detection limit for Δ*f* reached 0.8 GHz, determined by the standard errors when fitting the curves in Figure 2a. This corresponds to the yeast surface density of 400 cell/mm^2^, useful for evaluating microbes contained in liquid solutions using elaborate deposition methods [38]. We also note that our sensors can be used for detecting various biological specimens, including molds and bacteria, because of their dielectric nature [8,20].

## 4. Conclusions

In conclusion, we have successfully developed a novel THz FP microcavity biosensor that incorporates a porous PTFE film with a dielectric constant close to unity. The deposition of the yeast layer on the PTFE film caused a redshift in the frequency, which is attributed to the increased optical path length inside the cavity. Our experimental results are supported by FDTD simulations, which confirm the importance of field localization and cavity enhancement for optimal sensing performance. We have established an explicit relationship between Δ*f* and sensing parameters, such as the index, thickness, and vertical location of the substance. THz imaging enabled us to map the spatial distribution of microbes using the explicit relationship between Δ*f* and the amount of microbes. By using PTFE films with dielectric constants close to unity, it is possible to locate the target substances towards the center of the microcavity where the field enhancement is strongest, leading to optimal sensitivity. We emphasize the reusability of our FP sensor device; in addition, the PTFE membrane is also reusable by applying a proper fungicide solution. Our study highlights the potential of this biosensing platform for the detection of biological substances, including hazardous pathogens, and contributes to the development of cost-effective large-area THz biosensors.

## Data Availability

Not applicable.
